# Indicator analysis of the footwork in the world's top badminton athletes based on markerless motion measurement

**DOI:** 10.3389/fspor.2026.1753118

**Published:** 2026-04-02

**Authors:** Hidehiko Shishido, Takeshi Nishijima

**Affiliations:** 1 Soka University, Hachioji, Tokyo, Japan; 2 Tokyo Metropolitan University, Hachioji, Tokyo, Japan

**Keywords:** badminton performance analysis, markerless motion capture, footwork speed, elite athletes, youth athlete development

## Abstract

**Introduction:**

This study aimed to quantitatively compare the footwork characteristics of world-class badminton players and high school athletes using a markerless motion measurement technique, and to clarify the effects of rally length on movement speed and speed variability.

**Methods:**

Positional data of players were automatically extracted from match footage, and multiple performance indicators, including movement speed, the coefficient of variation (CV) of speed, and the frequency of acceleration and deceleration – were calculated. Additionally, rallies were categorized into segments, and changes in footwork characteristics across these segments were analyzed.

**Results:**

The results revealed high speed variability accompanied by rapid acceleration and deceleration in the early stages of rallies, particularly pronounced in top-level players who exhibited large speed fluctuations over short periods. As rallies progressed, both average speed and speed variability gradually decreased, suggesting that players may efficiently regulate their pace to maintain performance. In high school athletes, maintaining speed variability during later rally stages was more challenging, likely reflecting limitations in physical capacity and technical skills.

**Discussion:**

These findings highlight not only the quantitative assessment of footwork but also the importance of qualitative aspects such as pace-control ability. The results provide valuable insights for designing training programs aimed at improving competitive performance.

## 1 Introduction

Footwork constitutes a fundamental determinant of performance in racket sports such as tennis and badminton (1–5). These sports are characterized by non-cyclical movements involving repeated explosive accelerations, abrupt stops, and precise directional changes within the confined space of the court (6, 7). Badminton, recognized as the fastest racket sport, requires the ability to move swiftly and efficiently across the court, a decisive factor in determining the match outcome. Superior footwork enables players to reach the optimal hitting point, thereby maximizing shot quality and power (8). Conversely, inefficient footwork, characterized by unnecessary movements and extra steps or accelerations, increases energy expenditure, leads to a loss of balance and more unforced errors, and reduces the ability to execute high-quality shots, thereby markedly impairing multiple aspects of performance. Footwork encompasses not only the capacity to move quickly but also preparatory movements for subsequent shots, as well as the ability to anticipate the opponent's actions to adjust positioning efficiently. Therefore, enhancing footwork efficiency is crucial for players to adapt flexibly to dynamic match situations and establish a competitive advantage (7, 9).

Although the indispensable role of footwork in performance is widely acknowledged, objective analyses of footwork in elite badminton players remain a major challenge (6, 10, 11). Traditional coaching and performance evaluation rely primarily on subjective observations and qualitative assessments, lacking the objective and precise data necessary for a scientific understanding of elite performance. For further advancement of athletic development, it is essential to quantify and objectively evaluate the footwork metrics of top-level players.

Recent advances in digital technology and sports science have provided efficient solutions to the challenges of objective footwork analysis. This progress has evolved into sophisticated modern technologies such as marker-based motion capture systems and wearable sensors (12–15). Among these, the emergence of computer vision and markerless motion measurement systems offer an attractive alternative by enabling the large-scale and high-precision collection of datasets from standard video footage in a non-invasive manner (16–19). These technologies allow for a detailed analysis of performance in real-world environments without interfering with the athletes' natural movements (11, 20).

Contemporary sports science encompasses a wide variety of motion analysis tools, each with its distinct advantages and limitations. Marker-based optical systems are considered the gold standard for accuracy in controlled laboratory environments (13). However, they are expensive and impractical for use in real-world settings. Wearable sensor technologies have gained popularity because of their portability and ability to collect real-time data; however, their accuracy can be compromised by drifting over extended periods (21). Markerless computer vision systems incorporate artificial intelligence (AI) and deep learning algorithms to automatically track and analyze athlete movements from standard video footage without requiring markers (12, 13, 19). With minimal setup time and no physical burden on athletes, these systems are particularly suited for capturing natural movements in real-world environments such as competition venues. Nevertheless, their accuracy may be affected by factors such as video quality, lighting conditions, and occlusion of body parts from the camera's view (12, 22).

These technologies are complementary. While markerless systems capture complex movements under natural conditions, wearable sensors can provide crucial physiological data, such as heart rate and exercise intensity, which video analysis alone cannot capture. By integrating these diverse data streams using AI, a more comprehensive and multidimensional perspective on athletic performance can be achieved.

This study aimed to quantify and visualize the footwork performance of world-class badminton players. By applying a uniquely developed markerless motion measurement framework, this study systematically analyzed the distance covered, movement speed, and variations in footwork patterns during international competitions, thereby establishing clear performance indicators for each athlete. The core of this method is a technique that extracts the positional data of players' feet from video footage and converts them into real-world coordinates (23). Specifically, the algorithm automatically calculates the lowermost point of the body's center of mass and tracks its movement. This is achieved through a homography transformation that maps the two-dimensional court image plane into an undistorted overhead view of the real-world court. This approach enables automatic computation and accumulation of large-scale, high-precision performance data, providing objective indicators of elite-level footwork.

The critical role of footwork in determining badminton performance has long been established (24–26). Researchers have examined the relationships among footwork, agility, balance, and speed using agility drills (7, 9, 27–29). While these studies consistently demonstrate that dedicated footwork training improves key indicators, many are limited by small sample sizes and controlled experimental settings (27, 28, 30). Moreover, previous research has revealed distinct discrepancies between laboratory-based performance measures and actual competitive success (31, 32). For instance, although elite athletes exhibit a high aerobic capacity (VO_2_ max) and maximal running speed, these laboratory-derived indices are insufficient predictors of on-court success. This finding suggests that although general physical capacities are indispensable, they are not sufficient in isolation; the actual differentiating factor lies in how these physical attributes are applied in competition (31).

The proposed approach, which directly measures movement speed and distance covered during real tournament activities, addresses this limitation. Furthermore, consideration of sex differences is crucial in sports science and must be incorporated when analyzing athletic performance (33).

The markerless analysis method proposed in this study provides a groundbreaking foundation for the objective evaluation of footwork in elite badminton players (11, 15). By analyzing large-scale datasets automatically extracted from match footage, this approach introduces scientific and quantitative evidence for footwork evaluation, which has traditionally relied on subjective observations. Specifically, it is expected to quantify individual athletes' footwork in terms of the distance covered, mean speed, and instantaneous maximum speed, thereby elucidating the relationships among footwork indicators, playing styles, and tactical approaches. Moreover, by examining changes in footwork over the course of a match, this method is expected to provide an objective assessment of the impact of fatigue on performance. Additionally, the analysis of footwork patterns is expected to reveal the specific demands imposed by tactical strategies (6).

Such insights can provide practical data for optimizing coaching and training programs. Ultimately, this study sought to establish objective footwork indicators that not only open new avenues for performance analysis in badminton but also contribute directly to enhancing athletic ability. These indicators are expected to serve as valuable guidelines for future talent identification and the design of training regimens (31, 32, 34).

## 2 Methods

### 2.1 Preparation of video materials and identification of footwork segments

The basis for the analysis consisted of high-resolution match video files recorded at 1,920 × 1,080 pixels. From these videos, the rally segments in which footwork movements were clearly visible were identified. Specifically, frames from the moment the serve was executed to the point at which the shuttlecock landed on the court were manually selected. The identified footwork segments were then automatically extracted from the original video files as individual clips using a custom program in conjunction with the video-processing tool FFmpeg. This approach excluded irrelevant footage (e.g., periods of inactivity or breaks), thereby enhancing the efficiency and precision of the subsequent analysis.

### 2.2 Markerless measurement of players' distance covered

A method was established to measure the distance covered by badminton players with high precision using only match footage, without the need for markers or wearable sensors. This approach combines two key technologies: the object detection algorithm [You Only Look Once (YOLO)] and homography transformation.

First, the YOLOv11 deep learning-based object detection model was applied to all frames of the extracted footwork clips. YOLO can detect and classify objects in an image in a single inference. By dividing the image into grids, YOLO directly predicts the probability of object presence, class, and bounding box, representing the position and size of the object, for each grid cell. This single-pass process allows YOLO to perform fast inferences, making it well-suited for tracking rapid and continuous movements typical in sports footage (35).

YOLOv11, employed in this study, represents a substantial evolution from its predecessors YOLOv8 and YOLOv9. Architectural optimizations and improved training techniques have enhanced the detection accuracy of higher-resolution images, enabling a more precise delineation of object boundaries. Consequently, even players in dense scenes or at certain distances can be reliably detected. The general-purpose pretrained model was deemed sufficient for player recognition, and no additional training was performed.

The detection accuracy of YOLO can decrease under occlusion when multiple individuals appear within a single bounding box, causing the algorithm to misidentify them as a single object and lose track of a person. However, in singles badminton footage, players rarely overlap, thereby limiting the impact of occlusions on detection accuracy.

YOLO outputs a class label and confidence score for each detected object. For each frame, the bounding box with the highest confidence score was selected, and its bottom edge was used as the player's projected center of mass on the court. This point, which is vertically projected from the body's center of mass to the ground, most accurately represents the footwork movement.

Because the videos were captured at an oblique angle, the two-dimensional coordinates in the footage did not correspond directly to the actual positions on the court. To resolve this problem and calculate the precise distances covered, we applied a homography transformation based on the methodology established in a previous study (23). This technique converts image coordinates into real-world measurements by defining the correspondence between the four corners of the badminton court in real units (e.g., millimeters) and their pixel coordinates in the video. A projection matrix was computed from these four points and applied to the YOLO-detected player coordinates, transforming the image positions into top-down coordinates in the real court.

**Figure 1:**
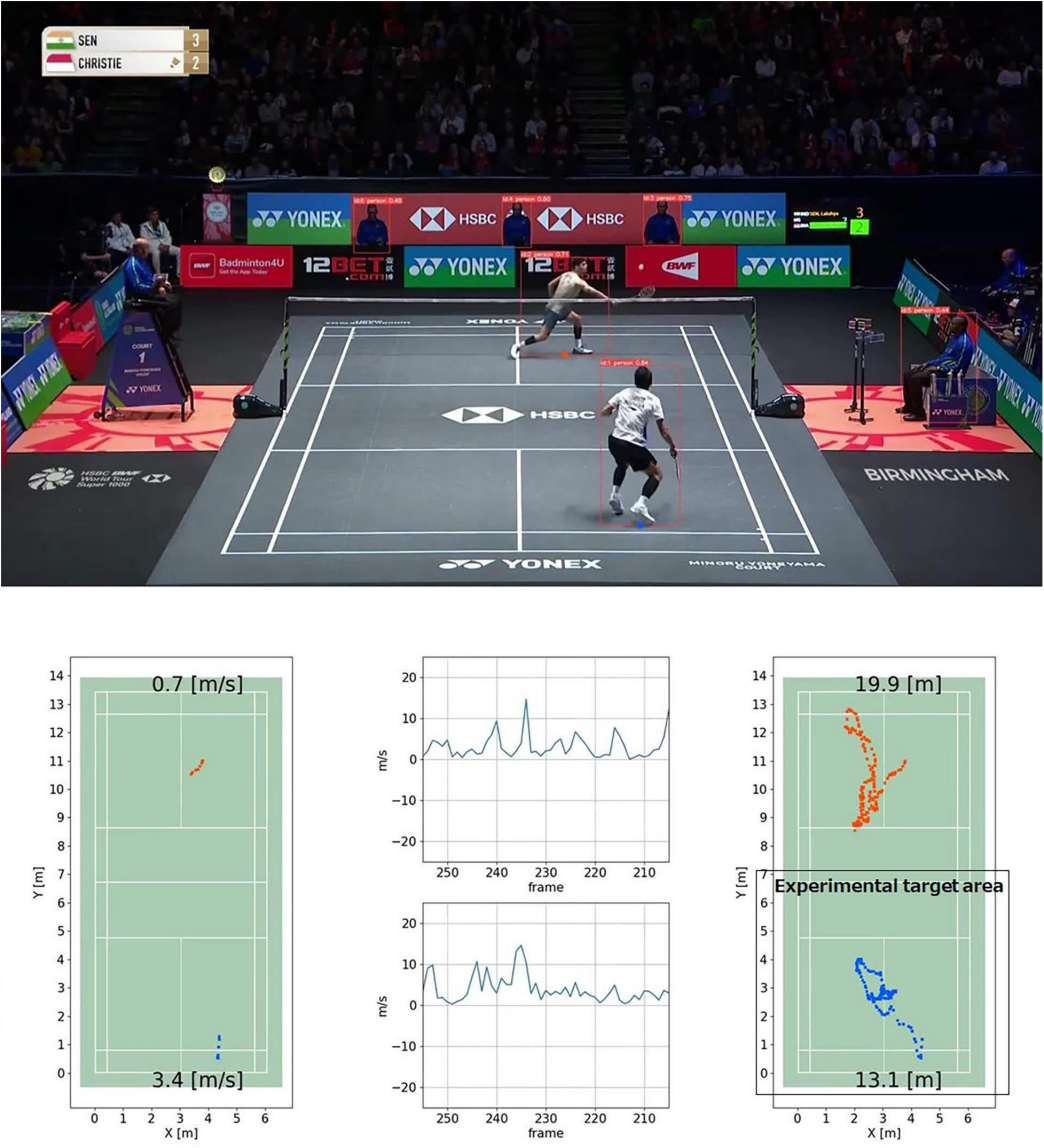
Upper section: the footage generated by applying YOLO to the match video. Lower-central section: The calculated movement speeds of the players. Lower-left section: The speeds of the player at the rear of the court and the player at the front of the court are plotted. Lower-right section: The total movement trajectories of both players. Source: Images were extracted from publicly available footage released by the Badminton World Federation (BWF) (36) and are used for research purposes.

This transformation yields precise real-world coordinates on the court, forming the basis for calculating physical distances and speeds. To illustrate the relationship before and after the transformation, Figure 1 compares the player positions in the original match footage with the corresponding positions on the top-down court map generated via homography. In previous validation (23), the accuracy of this transformation was reported as an average estimation error of 44.8 ± 22.6 mm, sufficient for footwork analysis. The accuracy differs between the near and far sides of the court; therefore, both sides cannot be analyzed using the same metric. Accordingly, this study focused on the near-side court area, which was consistent with the experimental region in (23), as shown in the lower-right panel of Figure 1.

### 2.3 Limitations of the analysis method

As described above, this study analyzed existing match footage by estimating top-down court coordinates through homography transformation and detecting players using YOLOv11. As noted in Section 2.2, homography transformation allows high-precision acquisition of player positions on the near side of the court, providing sufficient reliability for footwork analysis. However, this analysis was conducted using footage from standard match recordings rather than a controlled experimental environment with dedicated calibration markers, so geometric errors arising from camera angle and lens distortion were not eliminated. Nevertheless, in the analyzed footage, the court was centered in the frame and players remained within the court boundaries, which suggests that the influence of lens distortion, typically more pronounced at the image peripheries, was limited.

YOLOv11, compared with its predecessors YOLOv8 and YOLOv9, benefits from architectural optimizations and improved training techniques. These enhancements improve detection accuracy in high-resolution images and allow more precise delineation of object boundaries. As described in Section 2.2, occlusion between players is minimal in singles matches, which indicates that the impact of reduced detection accuracy due to occlusion is limited. Furthermore, by utilizing the existing pretrained model without additional training, the study avoided the complexity of further annotation while maintaining generalizability and reproducibility.

In contrast, when analyzing doubles matches, frequent player overlapping and extensive occlusion would inevitably reduce detection accuracy. In such cases, additional training focused on player regions or advanced tracking techniques would be practically essential. Although additional training could improve tracking accuracy, it requires substantial data preparation and annotation efforts, which creates a trade-off with model generalizability. Considering the characteristics of the target sport, this study therefore adopted the approach of using an existing pretrained model that provides sufficient accuracy for singles matches.

### 2.4 Calculation of footwork speed and organization of statistical data

Finally, the footwork speed was calculated using real-world coordinates obtained through homography transformation. The change in player position between consecutive frames was computed using the Euclidean distance. Specifically, if a player's coordinates in one frame are $\left(x_{1},y_{1}\right)$ and those in the subsequent frame are $\left(x_{2},y_{2}\right)$, the distance D traveled between frames is calculated as follows:

$$D=\sqrt{{\left(x_{2}-\;x_{1}\right)}^{2}+{\left(y_{2}-\;y_{1}\right)}^{2}}$$

The instantaneous speed for each frame (m/s) was obtained by dividing this distance by the time interval between frames. Given a video frame rate of 30 fps, this corresponds to approximately 0.03 s.

To reduce the potential noise in the instantaneous speed data and enhance reliability, we applied a moving average smoothing procedure. This process suppressed abrupt fluctuations in speed, allowing for a more accurate capture of gradual changes in footwork velocity.

For each clip, the mean and standard deviation of all speed data were calculated.

These statistical indicators objectively represent the velocity within each footwork segment. They were used for comparisons across multiple clips and players. The processed data were consolidated into summary files to prepare the dataset for detailed analysis of footwork characteristics.

### 2.5 Matches selected for analysis

This study analyzed publicly available footage of world championship matches, specifically men's and women's singles quarterfinals (eight matches: eight male and eight female players, 16 players in total), from the YONEX All England Open Badminton Championships 2024. The videos were obtained from official releases by the Badminton World Federation (36). All selected players were ranked among the top 30 in the world at the time of competition (37).

For comparison, four singles match each of male and female Japanese high school players (four male and four female players, eight players in total) were also analyzed. These videos were obtained from official releases of the Badminton Association of Japan (38). The selected high school players were the top finishers of domestic tournaments in Japan in 2023.

### 2.6 Data aggregation and visualization

In this study, data processing and graph generation were performed using Python to visualize the collected data and analyze the relationship between rally length and average movement speed. First, rally length was manually quantified by counting the number of shots per rally. Organizing data according to rally length allows footwork characteristics to be interpreted within the context of a rally. For instance, short rallies emphasize explosive power, whereas long rallies require endurance and strategic footwork; separating data by rally length enables the quantitative evaluation of these distinct abilities.

The aggregated data were then visualized using graphical tools. The proportion of rallies within each rally-length category was displayed as stacked bar graphs, providing a visual representation of the playing styles and tactical tendencies for each player or group (top athletes vs. high school players). For example, if top athletes exhibit a higher proportion of long rallies than high school players, this may indicate superior endurance and rally control. Such visualization is crucial not only for comparing movement speed but also for understanding the match contexts in which that speed is utilized.

Additionally, the average movement speed for each rally-length category was visualized using box-and-whisker plots. These plots succinctly represent data distribution, including media, quartiles, and outliers, facilitating comparisons between groups. By interpreting these plots, the analysis goes beyond simply identifying faster or slower players; it also reveals how the average movement speed changes as rallies progress, providing insights into footwork efficiency and endurance. Comparing the box-and-whisker plots of top athletes and high school players facilitates assessment of differences in speed across rally lengths. If the gap in average speed widens with longer rallies, it serves as objective evidence that top athletes possess footwork capable of sustaining higher-level rallies.

Through these visualization and interpretation techniques, it was possible to gain a deeper understanding of the individual strategies and physical capabilities that underlie footwork speed.

### 2.7 Statistical analysis

Statistical analyses were conducted to examine the presence of statistically significant differences in footwork speed between top athletes and high school players. Comparisons were performed within the same sex, and no analyses were conducted across sexes.

Footwork speed was assumed to be influenced by two factors: rally length within a single inning (e.g., 1–4 shots, 5–10 shots, 11–20 shots) and player category (top athletes vs. high school players). Therefore, a two-way analysis of variance (ANOVA) was applied. This method enables simultaneous evaluation of the main effects of rally length and player category, as well as their interaction, providing a multifaceted understanding of the factors that affect footwork speed.

Before testing, the data were assessed for normality and homogeneity of variance to ensure that the assumptions for ANOVA were met. The analysis used the mean movement speed data for each rally-length category within each group. The significance level was set at *p* < 0.05; differences with *p*-values below this threshold were considered statistically significant.

This statistical analysis was performed separately for the male and female datasets, comparing top athletes and high school players of each sex, thus comprehensively evaluating the differences in footwork speed between the two groups.

### 2.8 Calculation and analysis of the coefficient of variation (CV) of speed

In this study, the coefficient of variation (CV) of footwork speed was calculated to evaluate movement stability and acceleration-deceleration characteristics, which cannot be fully captured by the mean speed alone. The CV is a dimensionless index defined as the standard deviation of speed divided by the mean speed, as expressed by the following equation:

$$\mathrm{CV}=\frac{\mathrm{Standard}\;\mathrm{deviation}\;\mathrm{of}\;\mathrm{speed}}{\mathrm{Average}\;\mathrm{speed}\;}$$

While the average speed represents an absolute measure of movement level, the CV indicates the relative magnitude of variation with respect to that level. Therefore, it allows comparisons across players with different speed levels. Since this study targeted groups with different competitive levels, namely top athletes and high school players, comparisons based solely on average speed may not sufficiently capture the quality or stability of movement control. By using the CV, differences in speed levels are normalized, enabling its use as a surrogate indicator of the frequency of acceleration and deceleration and stability of movement control.

Badminton footwork involves non-cyclical movements with repeated rapid accelerations, decelerations, and directional changes. Large variations in speed may reflect frequent accelerations and decelerations or unnecessary movements, which can increase mechanical demand and reduce movement efficiency. Conversely, a low CV indicates that speed fluctuations are suppressed relative to the average speed, suggesting efficient and stable movement control. The purpose of this analysis was not to test statistically significant differences between groups, but rather to explore the existence of variability characteristics independent of average speed. Therefore, this index does not directly measure acceleration ability itself, but it serves as a complementary indicator for assessing acceleration-deceleration characteristics.

In this study, the average and standard deviation of instantaneous speed data were calculated for each rally segment, and the CV was computed for each player and rally-length category. Rally-length categories containing missing values (N.A.) were excluded from the analysis. The resulting CV data were organized into four categories: male top athletes, male high school players, female top athletes, and female high school players, and all players were included in the analysis.

No statistical hypothesis testing was performed for comparisons between groups. Instead, descriptive statistics and visualizations were used to examine trends. Box-and-whisker plots were employed for visualization, with rally length on the horizontal axis and CV on the vertical axis, showing the distribution of each category within each rally-length range. This approach allows a visual comparison of speed control stability and individual differences, which cannot be fully captured by average speed alone.

The introduction of this index provides a framework for evaluating footwork characteristics from multiple perspectives, capturing not only the quantitative aspect of “how fast a player moves” but also the qualitative aspect of “how stably that speed is controlled.” It serves as a complementary measure for understanding the efficient movement control of top athletes and provides a foundation for interpreting differences between high school players and elite athletes in terms of mechanical demands.

### 2.9 Calculation of total distance per rally and evaluation of load characteristics

In addition to the analysis of footwork speed, this study calculated the total distance traveled per rally as a distance-based metric to evaluate the characteristics of physical load during match progression. While speed represents the movement capacity per unit time and reflects explosive power and movement efficiency, the total amount of physical effort required by a player to cover the court cannot be fully captured by speed alone. Therefore, the total distance traveled was quantified and positioned as a fundamental load metric that complements speed-based indicators.

The total distance was calculated using the court coordinate data obtained after homography transformation described in Section 2.4. For each rally, the distance between consecutive frames was accumulated to determine the total distance (in meters) a player moved until scoring a point. This metric reflects the movement demand on the court directly, rather than the speed of movement itself.

Furthermore, based on the sequence of points in the match, all rallies were divided into three phases: early, middle, and late. This division was introduced to indirectly assess the accumulation of fatigue and changes in tactical development as the match progressed. The total distance for all rallies in each phase was aggregated, and the average and standard deviation were calculated.

Visualization was performed using bar plots with error bars, showing the average total distance (±standard deviation) for each phase. This approach allows visual comparison of trends in movement volume across match progression, differences in load distribution between top athletes and high school players, and the ability to maintain movement in the late phase.

The introduction of a distance-based metric provides a more multifaceted perspective for interpreting average speed. For example, a high average speed accompanied by an excessive total distance may indicate redundant movements rather than efficient positioning. Conversely, maintaining speed while limiting total distance may suggest high predictive ability or tactical efficiency.

Therefore, this metric provides a fundamental framework for evaluating the physical load characteristics of footwork and enables a more comprehensive understanding of the relationship between match progression, physiological demands, and player level.

### 2.10 Visualization of court occupancy heat maps and footwork by rally outcome

In addition to the quantitative comparison of footwork speed, this study visually examined players' spatial positioning and its relationship with rallying outcomes. Footwork is not only characterized by the amount of movement, such as speed and distance, but also by the spatial aspect of where players spend time on the court. Therefore, this supplementary analysis aimed to visualize positional distributions on the court and to complementarily examine differences in behavioral characteristics between top athletes and high school players. By classifying data according to rally outcomes, it was also possible to explore the relationship between spatial footwork characteristics and performance results.

This analysis used the real-world coordinate data (x, y) obtained through homography transformation as described in Section 2.2. The visualization was performed on a badminton court diagram based on actual dimensions (6,700 mm in length × 6,100 mm in width), and all frame-by-frame player position data were overlaid. To visualize the positional distribution, kernel density estimation (KDE) implemented in Python (matplotlib, seaborn) was applied. This approach smoothed the discrete coordinate data into a continuous density distribution, producing a heat map that highlights areas where players spent more time. The resulting maps allowed visual assessment of positional concentration and spatial spread on the court. In this analysis, no zone-based quantification of occupancy or statistical testing was performed, and the evaluation was based solely on descriptive visualization of positional distributions.

Furthermore, rallies were classified into winning and losing rallies, and separate heat maps were created for each outcome for four categories: male top athletes, male high school players, female top athletes, and female high school players. Rally outcomes were determined based on the official match score progression, and all frame-by-frame position data corresponding to each condition were aggregated for visualization. This procedure enabled visual comparison of spatial behavior characteristics between winning and losing rallies.

This visualization presents spatial characteristics of footwork that cannot be captured by average speed or total distance alone. It serves as a complementary material to evaluate qualitative aspects of footwork. By highlighting differences in spatial distribution, it provides fundamental data that may suggest positioning tendencies related to performance outcomes.

## 3 Statistical results

### 3.1 Analysis of footwork movement speed in male players

**Figure 2:**
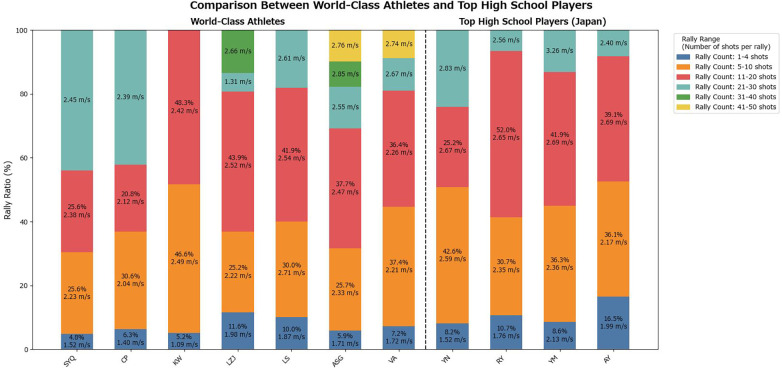
Proportion of rally counts per inning for male players.

**Figure 3:**
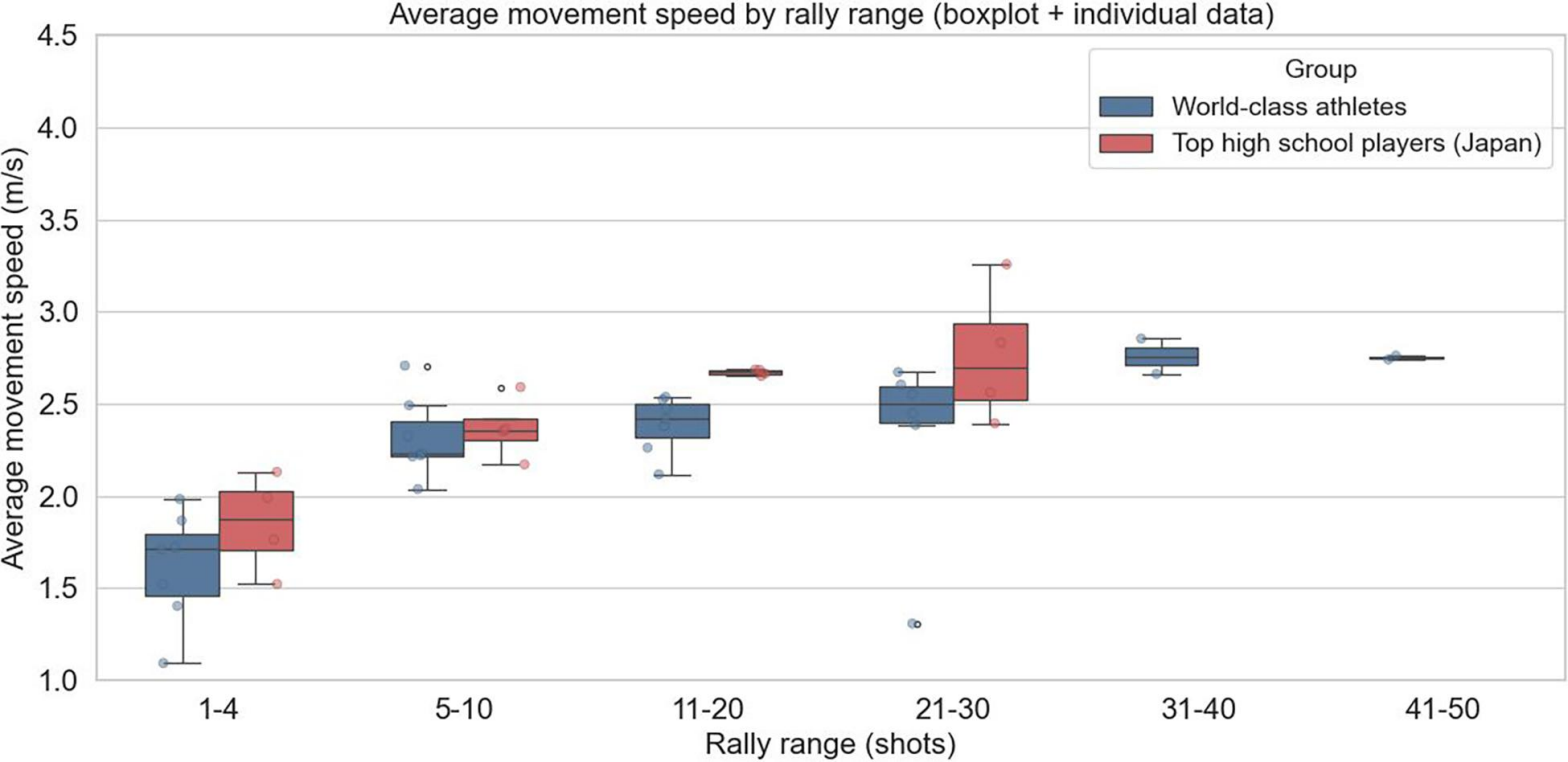
Average movement speed per rally-length category per inning for male players.

The distribution of rally lengths per inning for male players is shown in Figure 2. The mean movement speed for each rally-length category per inning is presented in Figure 3.

A two-way ANOVA was conducted on the male players' footwork movement speed, with player category and rally length as factors. The results revealed a statistically significant main effect of player category [*F*(1, 3) = 10.19, *p* = 0.0496], indicating that the mean footwork speed differed significantly between the top athletes and high school players. Additionally, the main effect of rally length was statistically significant [*F*(3, 3) = 23.38, *p* = 0.0139], demonstrating that footwork speed varied significantly depending on rally length.

### 3.2 Analysis of footwork movement speed in female players

**Figure 4:**
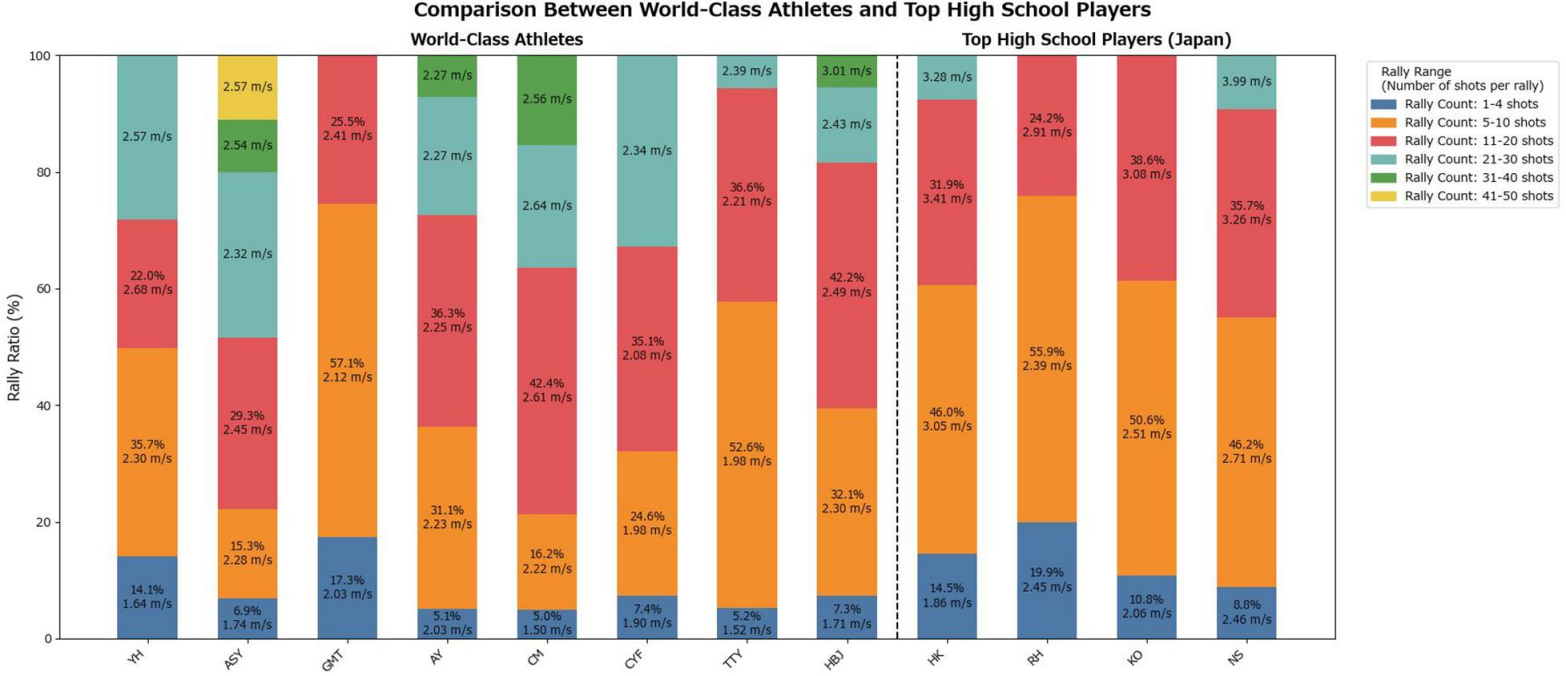
Proportion of rally counts per inning for female players.

**Figure 5:**
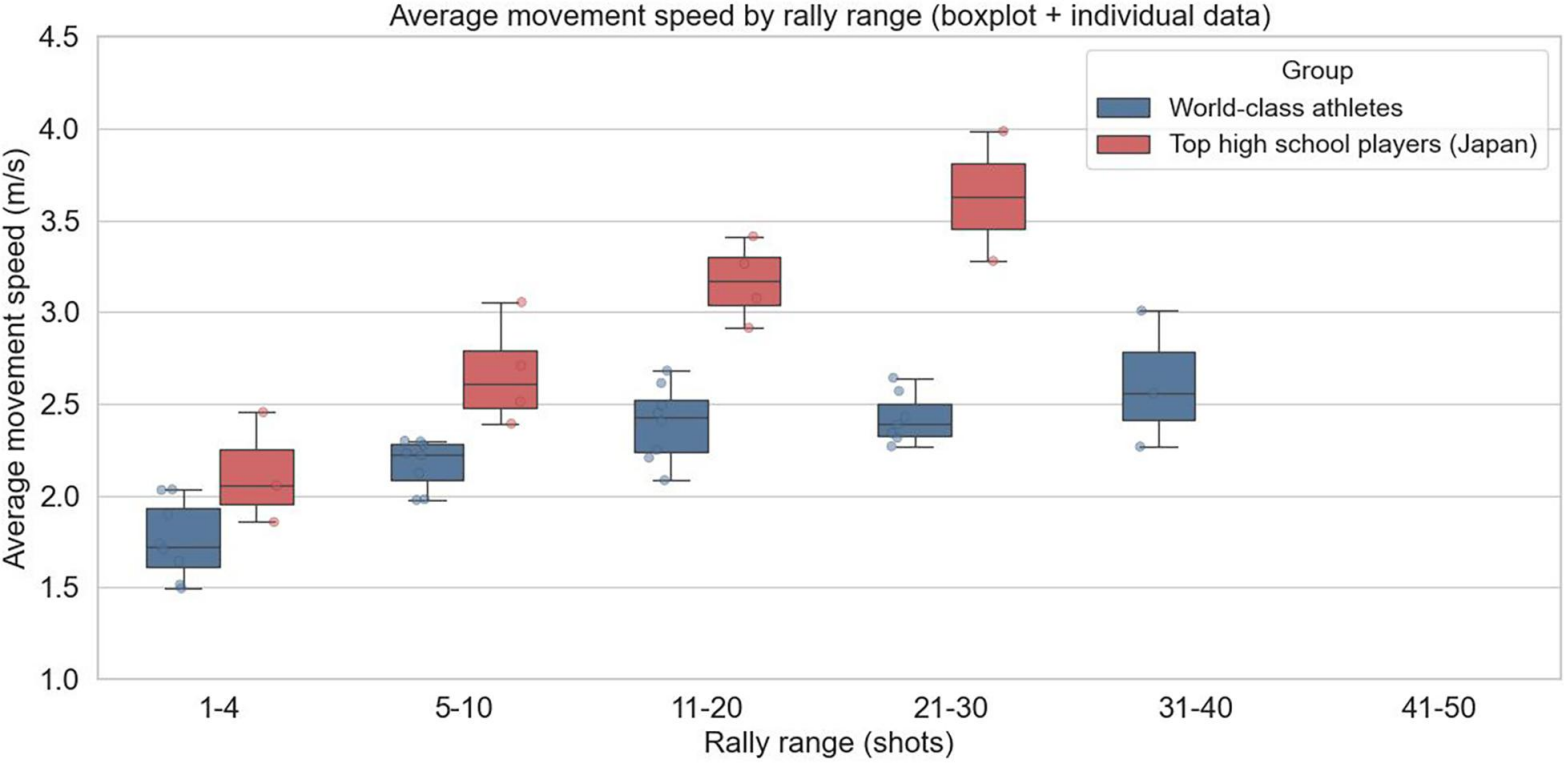
Average movement speed per rally-length category per inning for female players.

The distribution of rally lengths per inning for female players is shown in Figure 4. The mean movement speed for each rally-length category per inning is presented in Figure 5.

A two-way ANOVA was conducted on the female players' footwork movement speed, with player category and rally length as factors. Owing to limited data availability, the analysis was restricted to three rally-length categories with minimal missing data: 1–4 shots, 5–10 shots, and 11–20 shots. The results revealed a statistically significant main effect of player category [*F*(1, 2) = 32.13, *p* = 0.0297], indicating that the mean footwork speed differed significantly between top athletes and high school players. Additionally, the main effect of rally length was statistically significant [*F*(2, 2) = 21.25, *p* = 0.0449], demonstrating that footwork speed varied significantly depending on rally length.

## 4 Results and discussion

### 4.1 Comparative analysis of footwork speed by player category and rally range

This study compared the characteristics of footwork speed between top-level badminton players and high school players, while analyzing the influence of rally length on footwork from multiple perspectives. This analysis validates the utility of the markerless analysis method introduced in the Introduction. By leveraging the vast amount of data automatically extracted from match footage, this study provides scientific and quantitative evidence to support footwork evaluations that were previously largely subjective.

The results of the two-way ANOVA indicated that the player category (top athletes vs. high school players) and rally length had statistically significant effects on footwork speed. For male and female players, mean footwork speed differed significantly between the top athletes and high school players (male: *p* = 0.0496; female: *p* = 0.0297). These findings confirm that top athletes consistently maintain higher footwork speeds than high school players. This difference reflects not only raw speed but also multiple contributing factors, including reaction time to the shuttle, explosive acceleration, and anticipatory abilities, which collectively define footwork performance.

The simple hypothesis that footwork speed declines as rallies lengthen was not supported. In both sexes, the main effect of rally length was significant (male: *p* = 0.0139; female: *p* = 0.0449), demonstrating that footwork speed varies depending on rally length. Top athletes maintained relatively stable speeds of approximately 2.2–2.6 m/s even during long rallies, suggesting an ability to sustain performance under fatigue through efficient movement. By contrast, high school players occasionally record apparent speeds that exceeded the average speed of top athletes in longer rallies. This result is likely not due to efficient footwork but rather reflects inefficient, unnecessary movements arising from a lack of composure during extended rallies, which temporarily inflate the observed speed. Thus, using average footwork speed as a quantitative metric allows for an objective assessment of how fatigue differentially affects distinct player groups.

Furthermore, this study provides a foundation for linking footwork metrics to individual playing styles and tactical tendencies. Top athletes experienced rallies ranging from 1 to 50 shots, with a concentration in the 11–20 shot range, indicating their ability to adapt to a wide variety of rally scenarios. In contrast, the high school players' rallies were concentrated in the 5–10 shot range, with few long rallies. This difference underscores the fact that top athletes can effectively use their endurance, technical skills, and tactical knowledge to conclude points successfully, even in extended rallies. These analyses provide practical data for optimizing coaching strategies and training programs.

In conclusion, this study demonstrates that the markerless analysis method is a powerful tool for the scientific and quantitative evaluation of footwork, objective assessment of fatigue effects, and elucidation of the relationship between footwork and playing style. Thus, the objectives outlined in the Introduction were achieved. Future analyses that incorporate more detailed metrics, such as directional changes and acceleration/deceleration frequency, may further elucidate the efficiency and adaptability underlying the exceptional performance of top athletes.

### 4.2 Comparison of average coefficient of variation by rally range

**Figure 6:**
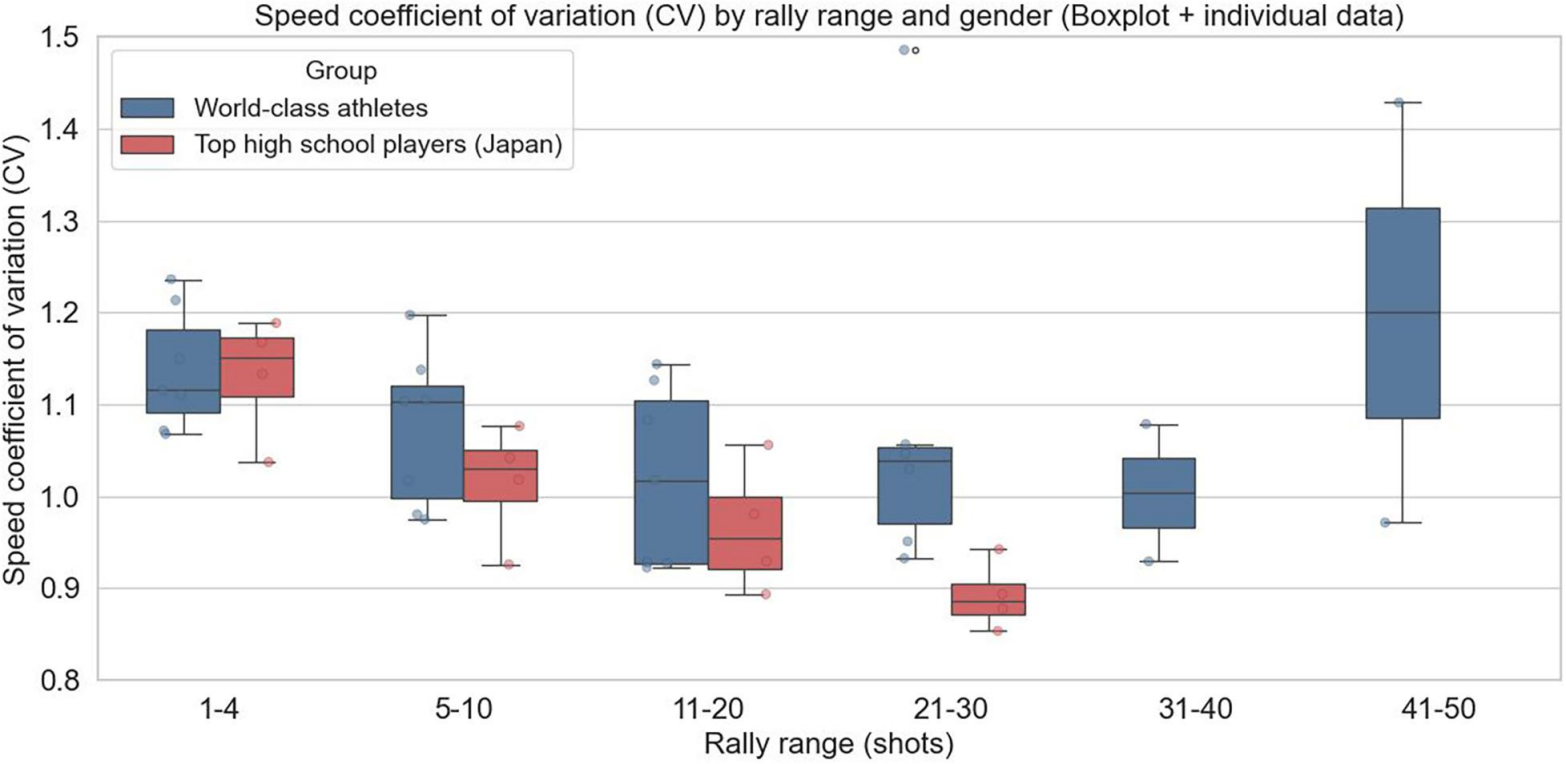
Average coefficient of variation (CV) of speed by rally range in male players (boxplot with individual data).

As shown in Figure 6, in male players, both high school students and top athletes exhibited high average coefficients of variation (CV) in the early phase of rallies (rallies 1–4). A high CV indicates large changes in speed over a short period, reflecting frequent rapid accelerations and decelerations. In the early stage of rallies, such active speed changes may occur as players attempt to gain initiative. In high school players, CV gradually decreased as the number of rally shots increased, and many instances in rallies 21–30 fell below 1.0. This suggests that as rallies prolong, the frequency and intensity of accelerations and decelerations are suppressed, and play may become more passive. In contrast, top athletes maintained a CV around 1.0 even in the middle to late stages of rallies, indicating sustained control of accelerations and decelerations during longer rallies. The maintained distribution width in the boxplots suggests that speed changes were actively adjusted according to situational demands.

**Figure 7:**
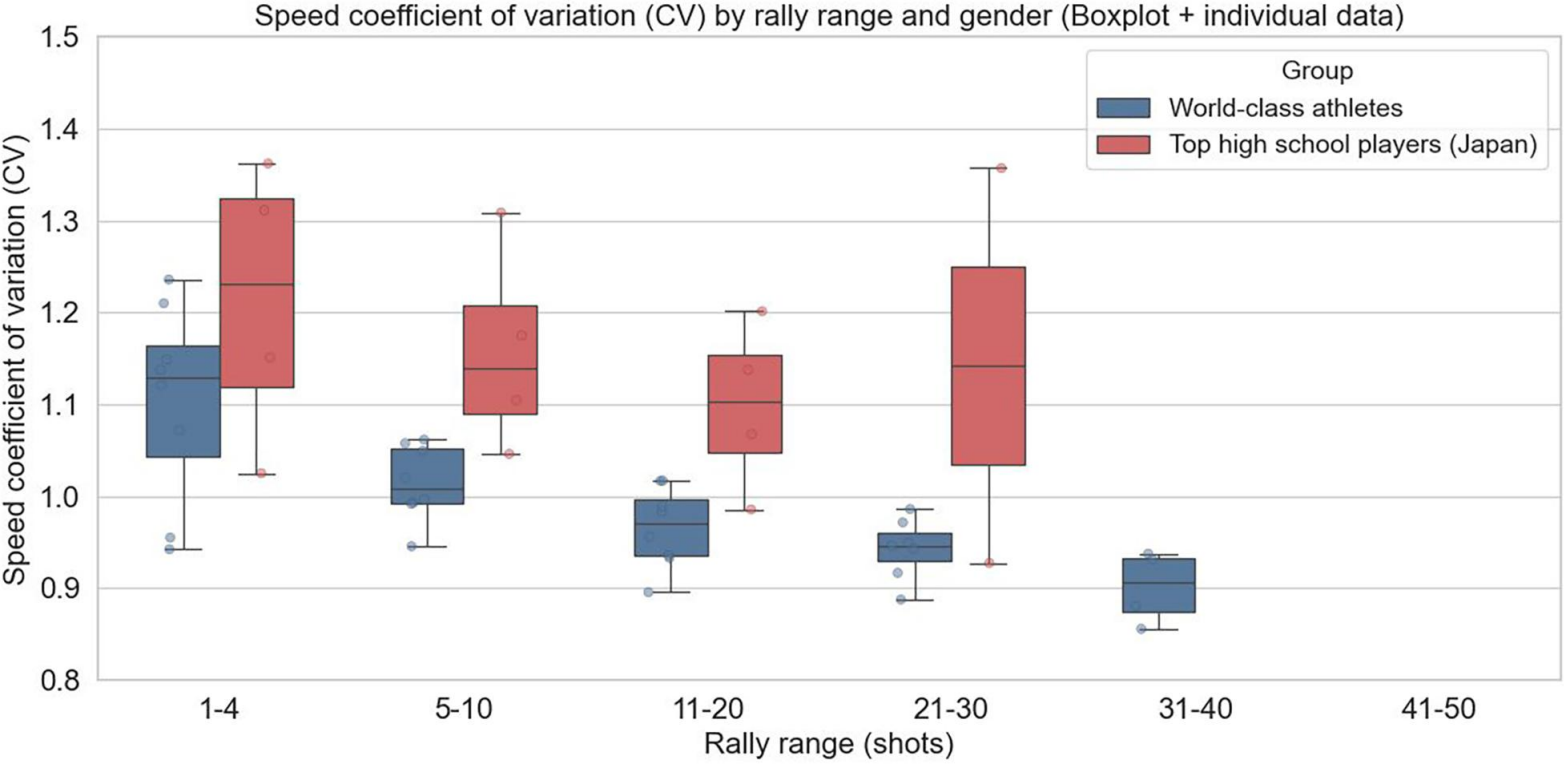
Average coefficient of variation (CV) of speed by rally range in female players (boxplot with individual data).

In female players (Figure 7), high school students showed generally high CVs throughout, particularly in the early phase of rallies, suggesting frequent rapid accelerations and decelerations and insufficiently stabilized movements. In top athletes, CV tended to decrease gradually as rallies progressed. This indicates that during longer rallies, unnecessary accelerations and decelerations were suppressed, while speed changes were generated only in necessary situations. Excessive accelerations and decelerations increase energy expenditure, so high-level players are likely to control speed changes over the entire rally in a strategically efficient manner.

Thus, the CV used in this study should not be interpreted merely as a measure of speed variability, but rather as a comprehensive indicator reflecting the frequency and intensity of accelerations and decelerations during rallies. Differences in competitive level may manifest as the ability to intentionally and efficiently control accelerations and decelerations. Large speed variations can involve frequent accelerations, decelerations, or unnecessary steps, increasing mechanical load and energy expenditure. Therefore, the difficulty high school players have in maintaining stable CV in the later phases of rallies may also contribute to differences in performance from the perspective of movement efficiency.

### 4.3 Comparison of average movement distance by rally phase

**Figure 8:**
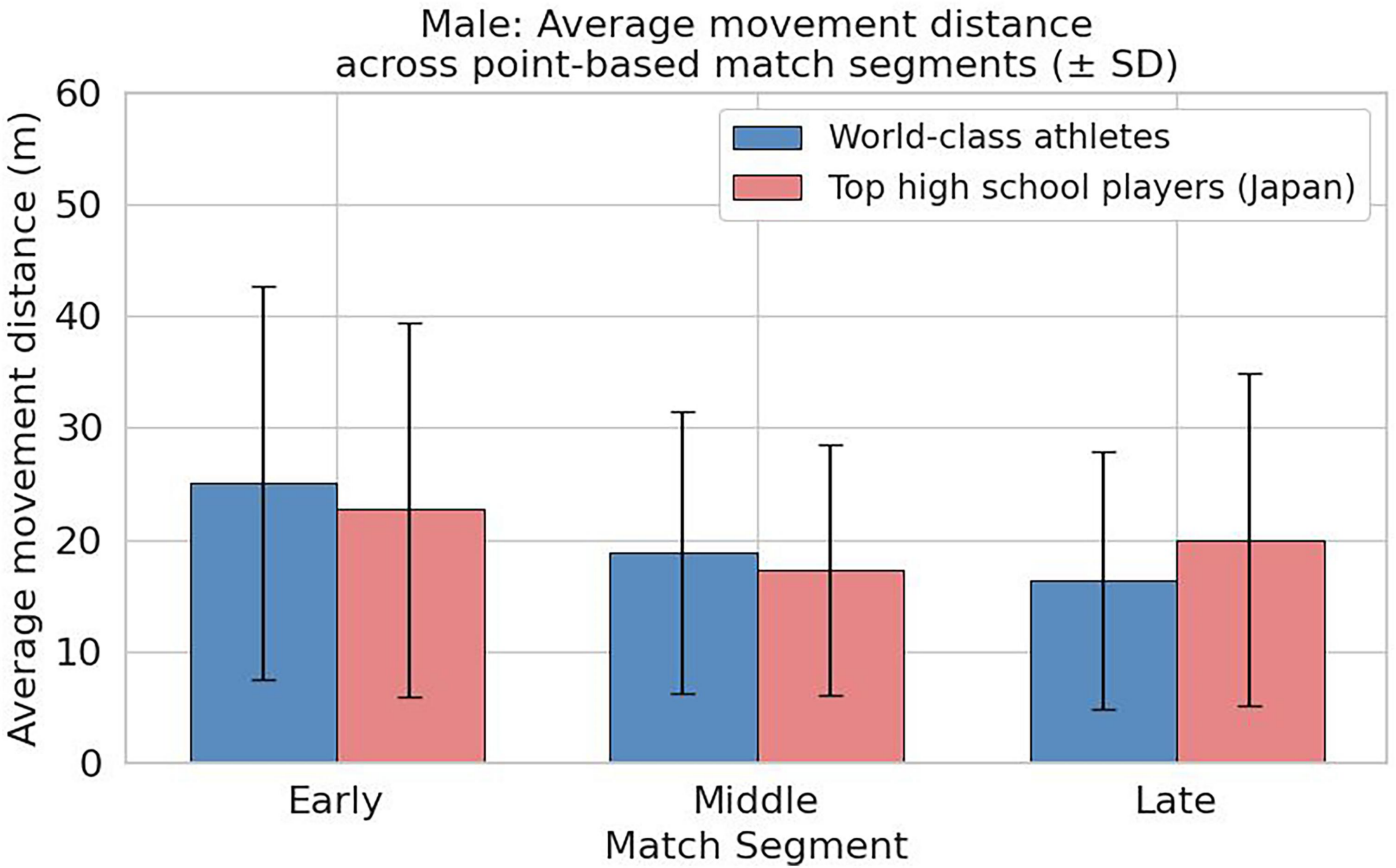
Comparison of average movement distance by rally phase (early, middle, late) in male players (high school vs. top athletes).

As shown in Figure 8, in male players, high school students had average movement distances of 22.72 m in the early phase, 17.31 m in the middle phase, and 20.01 m in the late phase, with the lowest value occurring in the middle phase followed by a rebound in the late phase. In contrast, top athletes showed 25.04 m in the early phase, 18.83 m in the middle phase, and 16.34 m in the late phase, indicating a gradual decrease from the early to late phase. A decrease of approximately 8.7 m from the early to late phase was observed, demonstrating a different pattern compared to high school students. The standard deviation was largest in the early phase for both groups, reflecting substantial variability in movement distance at the start of rallies.

The gradual decrease observed in top athletes suggests that they were able to suppress unnecessary movement as rallies progressed, potentially reflecting optimized positioning and advanced anticipatory decision-making. The lowest values in the late phase indicate effective suppression of redundant movement and efficient court coverage. Conversely, the rebound in high school students during the late phase may indicate challenges in maintaining movement efficiency during prolonged rallies.

**Figure 9:**
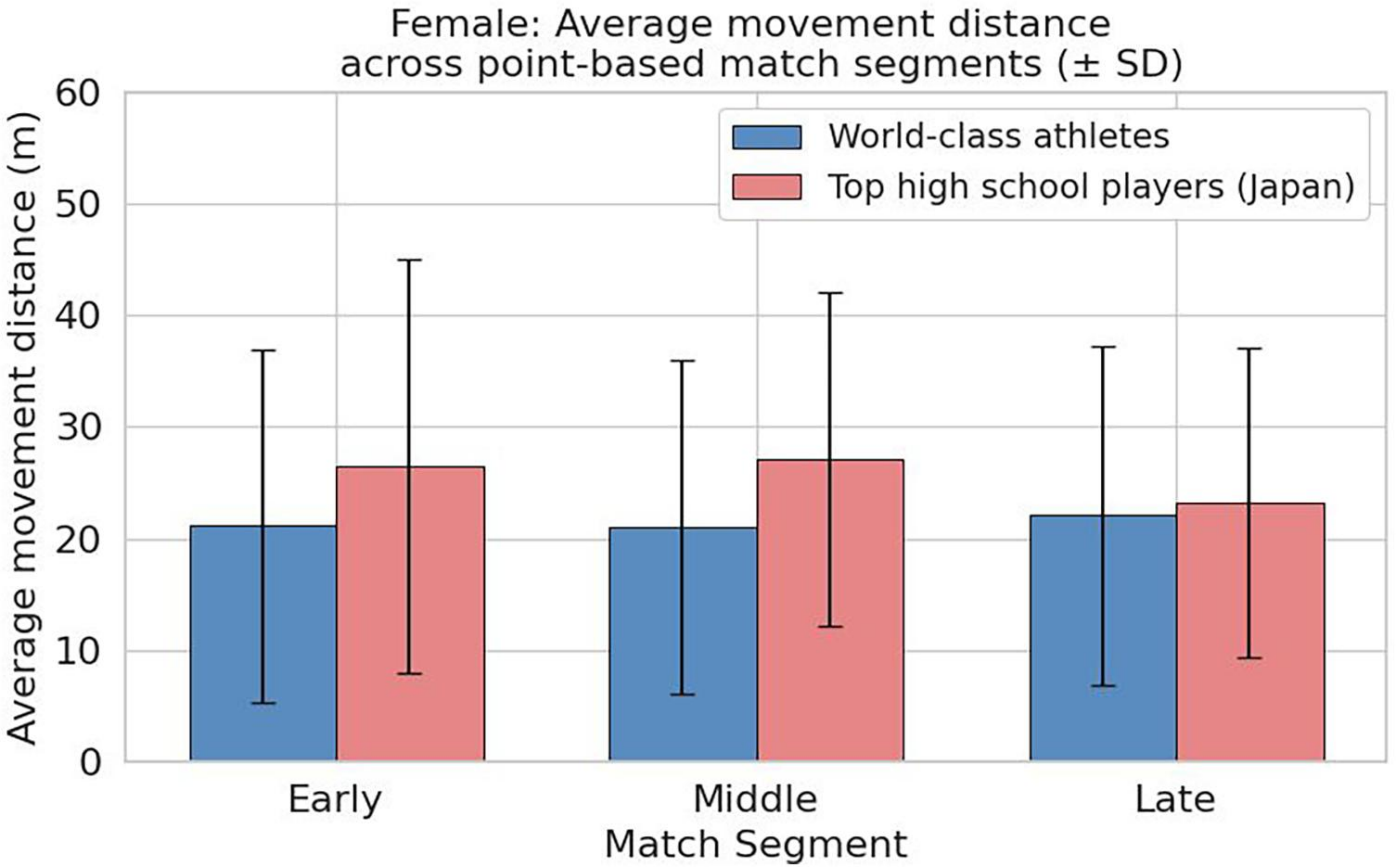
Comparison of average movement distance by rally phase (early, middle, late) in female players (high school vs. top athletes).

As shown in Figure 9, in female players, high school students had average movement distances of 26.48 m in the early phase, 27.09 m in the middle phase, and 23.17 m in the late phase, with the highest value in the middle phase. Movement increased from the early to middle phase and decreased by approximately 3.9 m in the late phase, likely to reflect increased movement demand due to more complex rally developments. Top athletes, on the other hand, maintained remarkably stable values, with 21.13 m in the early phase, 21.08 m in the middle phase, and 22.08 m in the late phase, with differences among phases remaining within approximately 1 m. Standard deviations also showed minimal variation between phases, indicating consistent movement distances.

These results quantitatively demonstrate that top athletes control their movement distances within a consistent range regardless of rally length, reflecting tactical stability and high energy efficiency in their play.

### 4.4 Spatial distribution of court footwork by rally outcome

**Figure 10:**
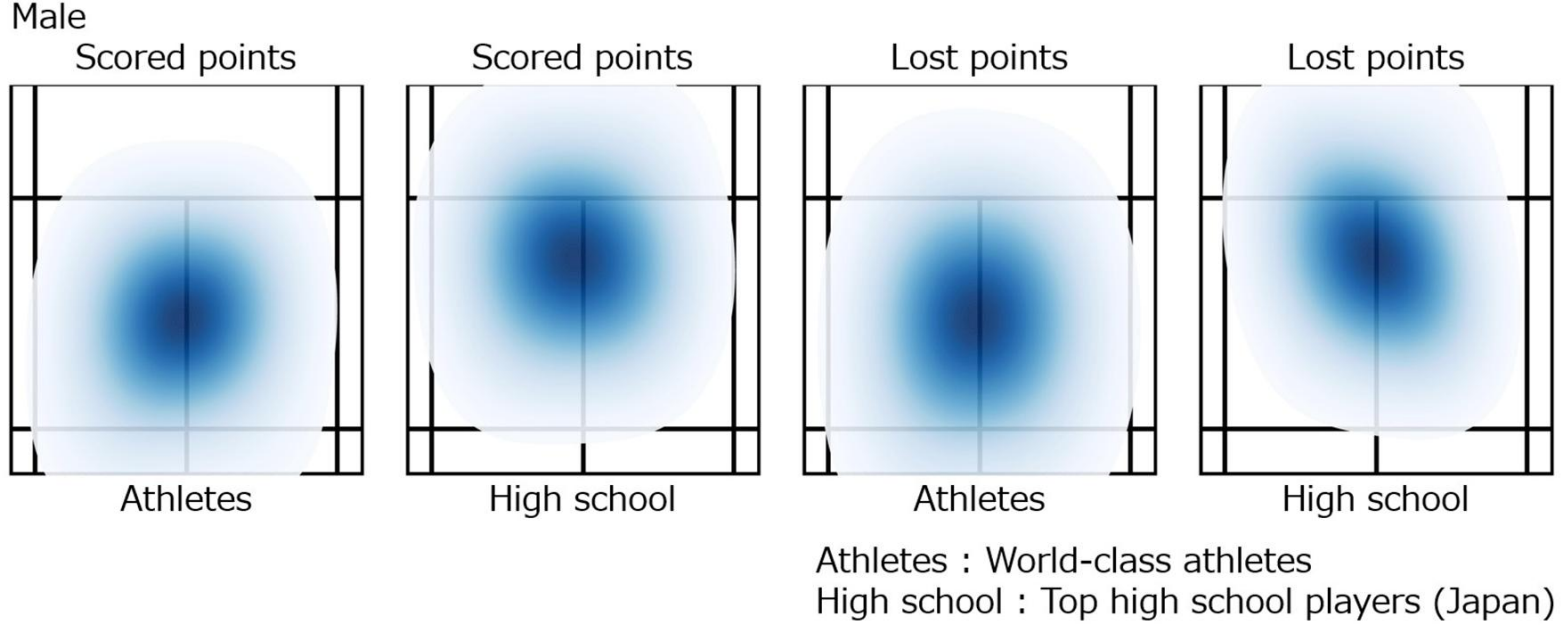
Spatial distribution of court footwork by rally outcome in male players (heatmap).

**Figure 11:**
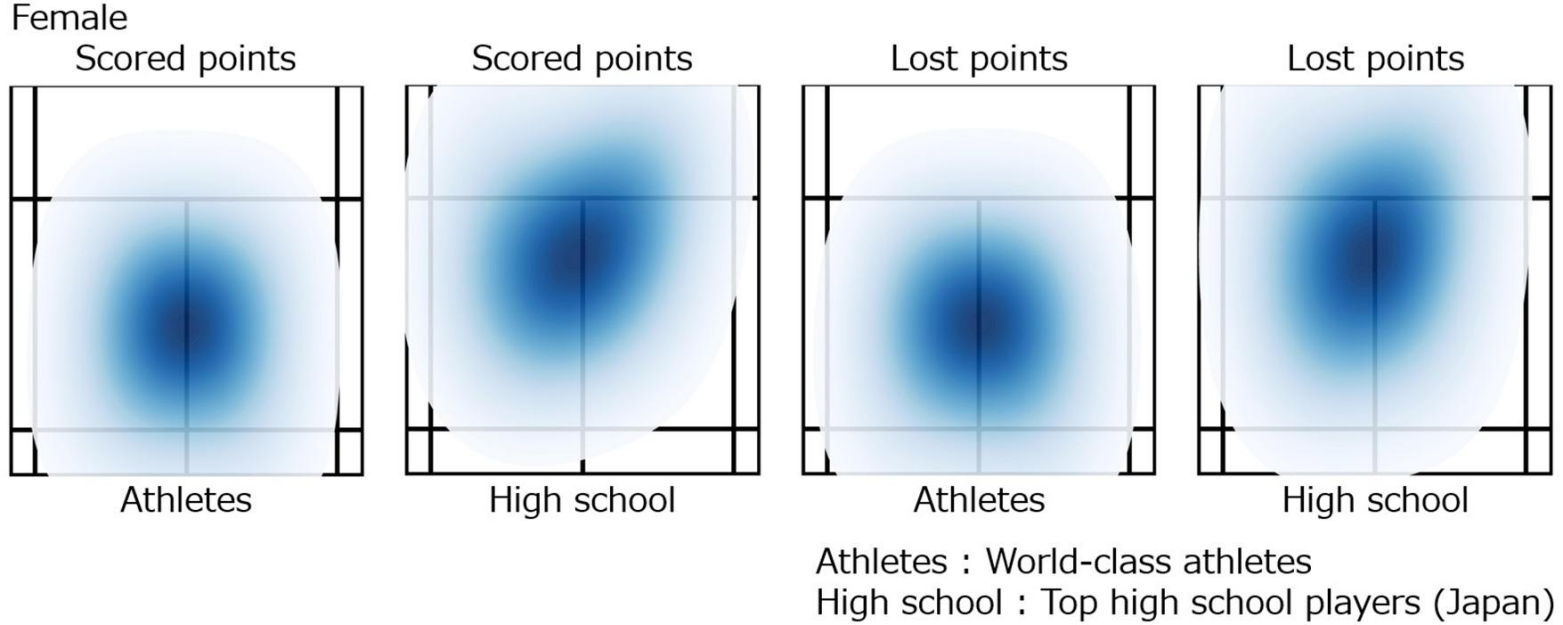
Spatial distribution of court footwork by rally outcome in female players (heatmap).

The results of the heatmap analysis are shown in Figure 10 for male players and Figure 11 for female players. In males, top athletes exhibited a symmetrical, nearly circular distribution centered on the court during scoring rallies. In contrast, high school students showed a peak approximately 1 m closer to the net than the court center, forming a slightly elongated oval shape along the front-back axis. While lost points, top athletes displayed a slight increase in front-back spread, but the distribution remained centered near the middle of the court. High school students, however, showed an asymmetric distribution, with the previously elongated front-back oval rotated approximately 45 degrees to the left.

Similar tendencies were observed in females. During scoring rallies, top athletes again showed a circular distribution centered on the court, while high school students exhibited an elongated oval distribution shifted toward the net, accompanied by a spread rotated approximately 45 degrees laterally. During lost points, top athletes demonstrated a slight increase in front-back spread, whereas high school students largely maintained the same elongated oval distribution as during scoring rallies.

These results indicate differences in positional stability according to competitive level. The center of the court allows easy movement in all directions, and a distribution centered around the middle is considered a rational configuration for quickly transitioning between offense and defense. The maintenance of a central position by top athletes regardless of rally outcome may reflect rapid recovery movements and stable situational judgment. In contrast, the forward bias and diagonal orientation observed in high school students suggest a tendency to favor movements in specific directions. Overall, maintaining a central base and symmetry in distribution appears characteristic of high-level players, whereas developing athletes may benefit from training aimed at stabilizing court positioning.

## 5 Conclusions

This study quantitatively compared the footwork characteristics of world-class badminton players and top Japanese high school players using a self-developed markerless motion analysis framework, based on multiple complementary indicators. Using actual coordinate data automatically extracted from match videos as the foundation, we integrated average movement speed, the coefficient of variation (CV) of speed, total movement distance per inning, and spatial indicators such as court occupancy heatmaps, thereby providing a framework to evaluate both the quantitative and qualitative aspects of footwork.

Analysis of average movement speed revealed main effects of player category and rally range in both male and female players, indicating that competitive level and rally length significantly influence footwork speed. Top athletes tended to maintain stable speeds even in extended rallies, whereas high school players sometimes exhibited apparent speed increases depending on rally conditions. This suggests differences not only in physical capacity but also in tactical leeway and movement efficiency.

The introduction of the coefficient of variation (CV) clarified qualitative aspects, such as the frequency and control characteristics of acceleration and deceleration. Among males, top athletes-maintained CV at a consistent level even in long rallies, indicating continuous control of acceleration and deceleration according to the situation. In contrast, high school players showed a tendency for CV to decrease as rallies progressed, suggesting a more passive play and reduced control of speed fluctuations. Among females, high school players generally exhibited high CV values, indicating challenges in movement stability, whereas top athletes showed a trend of suppressing CV as rallies progressed. These results demonstrate that, in addition to “how fast” a player moves, “how stably speed is controlled” is an important factor distinguishing competitive levels.

Analysis of total movement distance per inning revealed a gradual decrease for top athletes as matches progressed, suggesting optimization of spatial efficiency. The reduction in distance toward the end of matches may reflect predictive ability and high tactical rationality. In contrast, high school players tended to maintain or even increase total distance in the later stages, suggesting underdeveloped spatial control and unnecessary movement.

Moreover, visualization using court occupancy heatmaps and by rally outcome highlighted spatial footwork characteristics that cannot be captured by speed or distance alone. Top athletes showed rational area-specific occupancy during scoring rallies, while high school players exhibited more dispersed distributions. These spatial patterns provide foundational data that may relate to performance outcomes.

In summary, this study demonstrates that a multi-indicator integrated approach based on markerless analysis can objectively visualize elite players' footwork characteristics and quantitatively identify areas for improvement in high school players. By combining multiple perspectives—average speed, CV, total movement distance, and spatial distribution—the study provides a comprehensive framework for evaluating both the quantity and quality of footwork.

Since this method allows for the collection of large-scale data using only publicly available match videos, it holds potential for long-term performance tracking and tactical analysis in the future. Furthermore, integrating spatial indicators with physiological data may contribute to the development of a more comprehensive framework for assessing competitive performance.

## Data Availability

The raw data supporting the conclusions of this article will be made available by the authors, without undue reservation.
