# Correction: Indicator analysis of the footwork in the world's top badminton athletes based on markerless motion measurement

**DOI:** 10.3389/fspor.2026.1881930

**Published:** 2026-07-06

**Authors:** Hidehiko Shishido, Takeshi Nishijima

**Affiliations:** 1 Soka University, Hachioji, Tokyo, Japan; 2 Tokyo Metropolitan University, Hachioji, Tokyo, Japan

**Keywords:** badminton performance analysis, markerless motion capture, footwork speed, elite athletes, youth athlete development

Reference 29 was erroneously written as Dinç M, Yılmaz G, Şengür E. The effect of core training on speed, agility, strength and jumping performance in 10–12 year old female badminton players. *Pedagogy Phys Cul Sports*. (2025) 29(3):211–218. doi: 10.15561/26649837.2025.0307. It should be Kahraman, MZ, Kızılca S. The effect of core training on speed, agility, strength and jumping performance in 10–12 year old female badminton players. *Pedagogy Phys Cul Sports.* (2025) 29(3), 211–218. doi: 10.15561/26649837.2025.0307.

The original version of this article has been updated.

